# Supplementary material for: Sensory function and somatosensorial system changes according to visual acuity and throwing techniques in goalball players: A cross-sectional study
Source: PLoS One. 2024 Mar 13;19(3):e0296948. doi: 10.1371/journal.pone.0296948 (PMC10936811; doi:10.1371/journal.pone.0296948)
Supplement: Attachment — Submitted filename: PONE-D-23-29101_reviewers comments.pdf [file pone.0296948.s003.pdf]

PLOS ONE

Specific changes in sensory function and somatosensorial system according to visual acuity and throwing techniques in goalball players

--Manuscript Draft--

**Manuscript Number:**	PONE-D-23-29101
**Article Type:**	Research Article
**Full Title:**	Specific changes in sensory function and somatosensorial system according to visual acuity and throwing techniques in goalball players
**Short Title:**	Short title: Effects of visual acuity and throwing techniques in goalball players
**Corresponding Author:**	Ayşenur Gökşen, Assistant Professor Doctor<br>Tarsus University: Tarsus Universitesi<br>Mersin, Tarsus TURKEY
**Keywords:**	disability sport; blindness; sensory system; reaction times.
**Abstract:**	The somatosensory system, which is a complex personal sensory system, is a basic feature that makes an athlete an individual. This study aims to evaluate the sensory tests and sensory function according to the visual acuity and throwing techniques of male players interested in goalball. Goalball players who have different visual acuities (B1=6, B2=6, B3=5) participated in the study. The Somatosensorial System was evaluated with sensory tests, and the shoulder proprioception test (joint position sense). Sensory function was evaluated with the flamingo balance test, functional reach test, and pro-agility test. Moreover, the factors affecting the throwing techniques in goalball were examined.<br>Seventeen male players, those aged 20–30 (20.82±3.94 years) who have been professionally engaged in goalball for at least three years (58.76–37.87 months) participated. Shoulder internal rotation joint position sense and the flamingo balance test were found to be different in the group with B1 visual acuity than in the group with B3 visual acuity (p=0.042* and 0.028 respectively). There was no difference between groups with B1-B2 visual acuity (p=0.394 and p=0.065) and between groups with B2-B3 visual acuity (p = 0.792 and p = 0.931). There was no difference in the area of the groups in terms of sensory tests (p> 0.05).<br>It is recommended that players with better visual acuity improve their proprioceptive quality and postural control by supplementing exercises with their eyes closed.
**Order of Authors:**	Ayşenur Gökşen, Assistant Professor Doctor
	Gonca İnce, Associate Professor Doctor
**Additional Information:**	
**Question**	**Response**
**Financial Disclosure**<br><br>Enter a financial disclosure statement that describes the sources of funding for the work included in this submission. Review the submission guidelines for detailed requirements. View published research articles from *PLOS ONE* for specific examples.<br><br>This statement is required for submission and **will appear in the published article** if the submission is accepted. Please make sure it is accurate.	This work was supported by The Scientific and Technological Research Council of Turkiye under Grant 122C262. The funders had no role in study design, data collection and analysis, decision to publish, or preparation of the manuscript.

Powered by Editorial Manager® and ProduXion Manager® from Aries Systems Corporation

**Unfunded studies**<br>Enter: *The author(s) received no specific funding for this work.*<br><br>**Funded studies**<br>Enter a statement with the following details:<br>• Initials of the authors who received each award<br>• Grant numbers awarded to each author<br>• The full name of each funder<br>• URL of each funder website<br>• Did the sponsors or funders play any role in the study design, data collection and analysis, decision to publish, or preparation of the manuscript?<br>• **NO** - Include this sentence at the end of your statement: *The funders had no role in study design, data collection and analysis, decision to publish, or preparation of the manuscript.*<br>• **YES** - Specify the role(s) played.<br><br>* typeset	
**Competing Interests**<br><br>Use the instructions below to enter a competing interest statement for this submission. On behalf of all authors, disclose any competing interests that could be perceived to bias this work—acknowledging all financial support and any other relevant financial or non-financial competing interests.<br><br>This statement is required for submission and **will appear in the published article** if the submission is accepted. Please make sure it is accurate and that any funding sources listed in your Funding Information later in the submission form are also declared in your Financial Disclosure statement.<br><br>View published research articles from *PLOS ONE* for specific examples.	The authors have declared that no competing interests exist.

**NO authors have competing interests**<br><br>Enter: *The authors have declared that no competing interests exist.*<br><br>**Authors with competing interests**<br><br>Enter competing interest details beginning with this statement:<br><br>*I have read the journal's policy and the authors of this manuscript have the following competing interests: [insert competing interests here]*<br><br>* typeset	
**Ethics Statement**<br><br>Enter an ethics statement for this submission. This statement is required if the study involved:<br><br>• Human participants<br>• Human specimens or tissue<br>• Vertebrate animals or cephalopods<br>• Vertebrate embryos or tissues<br>• Field research<br><br>Write "N/A" if the submission does not require an ethics statement.<br><br>General guidance is provided below. Consult the submission guidelines for detailed instructions. **Make sure that all information entered here is included in the Methods section of the manuscript.**	Necessary permissions for the study were obtained from the Non-Invasive Clinical Research Ethics Committee of Cukurova University Faculty of Medicine. (Date: 02.12.2022, decision no. 50.). Written informed consent was obtained from all individual participants included in the study. Need for informed consent was not waived by the ethics committee. Authors did not have access to information that could identify individual participants during or after data collection. This cross-sectional study was conducted between 3 December 2022 and 3 May 2023. The study was carried out in accordance with the Declaration of Helsinki.

Powered by Editorial Manager® and ProduXion Manager® from Aries Systems Corporation

**Format for specific study types**<br><br>**Human Subject Research (involving human participants and/or tissue)**<br>• Give the name of the institutional review board or ethics committee that approved the study<br>• Include the approval number and/or a statement indicating approval of this research<br>• Indicate the form of consent obtained (written/oral) or the reason that consent was not obtained (e.g. the data were analyzed anonymously)<br><br>**Animal Research (involving vertebrate animals, embryos or tissues)**<br>• Provide the name of the Institutional Animal Care and Use Committee (IACUC) or other relevant ethics board that reviewed the study protocol, and indicate whether they approved this research or granted a formal waiver of ethical approval<br>• Include an approval number if one was obtained<br>• If the study involved *non-human primates*, add *additional details* about animal welfare and steps taken to ameliorate suffering<br>• If anesthesia, euthanasia, or any kind of animal sacrifice is part of the study, include briefly which substances and/or methods were applied<br><br>**Field Research**<br><br>Include the following details if this study involves the collection of plant, animal, or other materials from a natural setting:<br>• Field permit number<br>• Name of the institution or relevant body that granted permission	
**Data Availability**<br><br>Authors are required to make all data underlying the findings described fully available, without restriction, and from the time of publication. PLOS allows rare exceptions to address legal and ethical concerns. See the PLOS Data Policy and FAQ for detailed information.	Yes - all data are fully available without restriction

Powered by Editorial Manager® and ProduXion Manager® from Aries Systems Corporation

A Data Availability Statement describing where the data can be found is required at submission. Your answers to this question constitute the Data Availability Statement and **will be published in the article**, if accepted.<br><br>**Important:** Stating 'data available on request from the author' is not sufficient. If your data are only available upon request, select 'No' for the first question and explain your exceptional situation in the text box.<br><br>Do the authors confirm that all data underlying the findings described in their manuscript are fully available without restriction?	
**Describe where the data may be found in full sentences. If you are copying our sample text, replace any instances of XXX with the appropriate details.**<br><br>• If the data are **held or will be held in a public repository**, include URLs, accession numbers or DOIs. If this information will only be available after acceptance, indicate this by ticking the box below. For example: *All XXX files are available from the XXX database (accession number(s) XXX, XXX.*).<br>• If the data are all contained **within the manuscript and/or Supporting Information files**, enter the following: *All relevant data are within the manuscript and its Supporting Information files.*<br>• If neither of these applies but you are able to provide **details of access elsewhere**, with or without limitations, please do so. For example:<br><br>*Data cannot be shared publicly because of [XXX]. Data are available from the XXX Institutional Data Access / Ethics Committee (contact via XXX) for researchers who meet the criteria for access to confidential data.*<br><br>*The data underlying the results presented in the study are available from (include the name of the third party*	The data underlying the results presented in the study are available at the corresponding author.

Powered by Editorial Manager® and ProduXion Manager® from Aries Systems Corporation

and contact information or URL).<br>• This text is appropriate if the data are owned by a third party and authors do not have permission to share the data.<br><br>* typeset	
Additional data availability information:	

Powered by Editorial Manager® and ProduXion Manager® from Aries Systems Corporation

Manuscript
Click here to access/download;Manuscript;goalball articleee.docx

Specific changes in sensory function and somatosensorial system according to visual acuity and throwing techniques in goalball players

Short title: Effects of visual acuity and throwing techniques in goalball players

Ayşenur Gökşen[1*&], Gonca İNCE[2&]

[1] Department of Physiotherapy and Rehabilitation, Faculty of Health Sciences, Tarsus University, Mersin, Turkiye.

[2] Department of Coaching Education / Sport-Health Sciences, Faculty of Sports Sciences, Cukurova University, Adana, Turkiye

* Corresponding author

E-mail: aysenurgoksen@tarsus.edu.tr (AG)

[&] These authors contributed equally to this work.

Abstract

The somatosensory system, which is a complex personal sensory system, is a basic feature that makes an athlete an individual. This study aims to evaluate the sensory tests and sensory function according to the visual acuity and throwing techniques of male players interested in goalball. Goalball players who have different visual acuities (B1=6, B2=6, B3=5) participated in the study. The Somatosensorial System was evaluated with sensory tests, and the shoulder proprioception test (joint position sense). Sensory function was evaluated with the flamingo balance test, functional reach test, and pro-agility test. Moreover, the factors affecting the throwing techniques in goalball were examined.

Seventeen male players, those aged 20–30 (20.82±3.94 years) who have been professionally engaged in goalball for at least three years (58.76–37.87 months) participated. Shoulder internal rotation joint position sense and the flamingo balance test were found to be different in the group with B1 visual acuity than in the group with B3 visual acuity ($p=0.042^*$ and 0.028 respectively). There was no difference between groups with B1-B2 visual acuity ($p=0.394$ and $p=0.065$) and between groups with B2-B3 visual acuity ($p = 0.792$ and $p = 0.931$). There was no difference in the area of the groups in terms of sensory tests ($p> 0.05$).

It is recommended that players with better visual acuity improve their proprioceptive quality and postural control by supplementing exercises with their eyes closed.

Key words: Disability sport, blindness, sensory system, reaction times.

Introduction

Goalball is a paralympic sport widely played by visually impaired individuals. The somatosensory system, which is a complex personal sensory system, affects an athlete's success. The somatosensory system is a personal complex sensory system that includes many different sensory systems such as vision, hearing, joint position sense (proprioceptive sense), and tactile sensation [1]. The somatosensory system is a basic feature that makes an athlete an individual [2]. It is a complex personal sensory system, that affects an athlete's success [2, 3]. It is known that the sense of sight is the most important part of the somatosensory system [4]. The greatest data flow to the somatosensory system after the vision is provided by the sense of touch perceived from the joint receptors under the name of proprioception and the senses perceived from the muscles and tendons [5]. In the literature, reaction time and postural control are accepted as a measure of sensory function [6]. The time elapsed between the arrival of all these perceived stimuli in the relevant somatosensory cortex and the emergence of the desired motor skill is called the reaction time [7]. The sense of touch, where the movement is perceived first, the senses created by the movement on the joint, the processing of information in the somatosensory cortex, and the reaction process are effective in the athlete's ability to learn a motor skill and perform this skill with the best performance [7]. The factors affecting this process can be examined in two ways as sensory and motor. Sensory factors can be interpreted by the somatosensory system and motor factors by muscle strength. In this study, the factors affecting reaction time will be examined in a multifaceted way.

It is thought that perceptions other than vision related to the somatosensory system develop more in visually impaired individuals [8, 9]. Although proprioceptive senses and the sense of touch are important for all players, for visually impaired players , these senses are more important to perform the movement in the correct and appropriate pattern [10]. The contribution of joint position sense and tactile sense will increase to prevent deterioration in the

proprioceptive sense with loss of vision [9]. Grouping individuals according to visual acuity will more clearly reveal the effect of vision on the somatosensory system. This study aims to evaluate the proprioceptive sense of the shoulder joint, the sense of touch of the palm, branch year, and sensory function (postural control and reaction time) according to the visual acuity of male players interested in goalball sports. Another aim of the study is to investigate the sensory factors affecting the throwing techniques of the athlete.

Materials and Methods

This project was carried out as a descriptive study in the cross-sectional survey model. The non-probability purposive sampling method was used in the selection of the sample. In the purposeful sampling method, the participants were selected using the stratified sampling method (according to their visual acuity, B1–B2-B3).

Individuals

This study was carried out on male players with different visual acuities (B1, B2, and B3) between the ages of 20 and 30 who have been professionally involved in goalball for at least three years in XXX Region visually impaired sports clubs. According to the power analysis, in order for a significant difference to occur between 3 different visual acuity groups, 5 people in each visual acuity group were included in the study. A total of 15 male players were included in the study [11]. Necessary permissions for the study were obtained from the Non-Invasive Clinical Research Ethics Committee of XXX University Faculty of Medicine. (Date: 02.12.2022, decision no. 50.). Written informed consent was obtained from all individual participants included in the study. Need for informed consent was not waived by the ethics committee. Authors did not have access to information that could identify individual participants during or after data collection. This cross-sectional study was conducted between 3 December 2022 and 3 May 2023. The study was carried out in accordance with the Declaration of Helsinki.

Inclusion criteria for the study:

In this study, visually impaired male players aged 18–30 who have been playing on the goalball team for at least three years and who regularly practice goalball were included.

Exclusion criteria from the study:

- Patients with a deviation of more than 30 degrees according to the Fukuda balance test were not included in the study because it may affect the postural control results of the study.
- Players who experienced serious injury within the first 6 weeks after the start of the study were not included in the study.
- Players with health problems (e.g., cancer, arthritis, heart disease, lung disease, and neurological disease) were not included in the study.
- Players with a disability other than the visually impaired were not included in the study.
- Players who regularly engage in other sports branches, except for goalball, were not included in the study.

Demographic characteristics of the players included in the study, somatosensory system (shoulder joint proprioception sensation and sense of touch), postural control (static and dynamic), and reaction time were evaluated.

Demographic features

A demographic form was presented in which individuals were questioned about visual acuity, dominant extremity, age, body mass index, sports age, branch years, injury history, occupation, throwing techniques, and additional systemic diseases.

Evaluation of the somatosensory system

The somatosensory systems of the individuals included in the study were evaluated by measuring sensory tests which are shoulder joint proprioception sense and the tactile sense of the dominant hand. (Figure 1.)

Shoulder joint proprioception sense

The joint position sense during the internal and external rotation of the shoulder was evaluated by measuring in the supine position with an inclinometer device. The reliability rate of the inclinometer device for joint position sense is reported to be % 95-99 [12]. The target angle was determined to be 90% of joint mobility. The difference between the athlete's target angle and the angle she repositioned was recorded as the declination angle. The declination angle was used for statistical analysis.

Sensory tests

The sense of touch was evaluated under two different headings: light touch and two-point discrimination, from the shoulder joint and palmar aspect of the hand.

Tactile sense was evaluated with Semmes-Weinstein Monofilament test and two-point discrimination tests. Before the tests, a familiarity trial was conducted. During the Semmes-Weinstein monofilament test, dermatome sites on the hand were considered. The filament was pressed and held for 1 second until it bent laterally to the dermatome area to be evaluated. The athlete was asked if he felt the pressure of the monofilament. Each evaluation was made three times, and 15 seconds waited after each application. Verbal notifications from athletes recorded. 1 mm precision calliper (Duratech™ TA-2081) was used to evaluate the two-point discrimination**.** The midpoint of the palmar aspect of the hand was also marked to standardize the test site. For the palmar face of the hand, the line drawn at the midpoint of the palmar face of the hand is the target. With the calliper, it was applied with equal pressure from both ends. Athletes were instructed to report if they felt they had lost a point or two. Athletes were instructed to report if they felt a point or two. Evaluations were made on the dominant extremity [13].

Evaluation of Sensory Function

Sensory function was assessed by measuring reaction time and postural control.

Evaluation of reaction time

Reaction time was evaluated with the pro-agility test protocol, which was modified in accordance with the disability status and applied to all athletes by using an eye patch. A 20-meter test area was created by placing the funnels 5 yards (4.57 m) to the left and right of the starting line. A photocell gate was placed at the start line to obtain repetitive transit times. The athlete took his place at the starting line before the practice started. When she was ready, she touched the funnel on the right and then the funnel on the left, crossing the starting line and ending the test. The time seen in the photocell was recorded, see Figure 2.

Evaluation postural control

Postural control was evaluated statically and dynamically with the eyes closed.

Flamingo Balance Test

The Flamingo Balance Test is a test used to evaluate the static balance of individuals. The validity and reliability of the flamingo balance test made by Tsigilis et al [14]. A wooden block consisting of 50 cm long, 4 cm high, and 3 cm wide beams and 2 short, 15 cm long, 2 cm wide beams was used for the Flamengo balance test. The test was repeated until the athlete understood the test and followed the explanations correctly, and then the measurement test was started. The arms were spread out to the sides and used for balance. When the athlete was ready, the stopwatch was started by cutting the support, and time was stopped whenever the balance was disturbed. All players wore eye patches during the test.

Functional Reach Test

The athlete was asked to stand on the side of the wall on which a tape measure was affixed, with the arm of her choice on the side of the wall. The preferred arm was kept straight with the elbow at 180 degrees, and the arm was raised until 90 degrees of flexion was achieved at the

shoulder. The starting point of the longest finger was marked as the starting point of the individual. The individual reached the last point he could reach by leaning forward to follow the tape measure without disturbing the parallelism of the elbow, hand position, and arm with the ground. This end point was marked, and the difference with the starting point was recorded in centimetres (cm). The test was repeated three times, and the best value was recorded. All players wore eye patches during the test.

Statistical analysis

Data were analyzed using IBM SPSS Statistics ver. 24.0 (IBM Co., Armonk, NY, USA). The statistics of descriptive variables were reported using mean and standard deviation (SD). The normality of distribution was tested by the Kolmogorov–Smirnov test. Since the data were not normally distributed, the Mann Whitney u and Kruskal Wallis test was conducted to compare differences between the results. Effect sizes for Kruskal Wallis test were calculated as Cohen d recommendations: where 0.2–0.49 is a small effect, 0.5–0.79 is a moderate effect, and ≥0.8 is a large effect [10]. Based on a sample study, it was concluded that when the required sample size was calculated, a total of 15 players , ideally 5 for each group, should be evaluated [15]. Spearman correlation coefficient was used to establish relationships between parameters. For all analyses, the threshold for statistical significance was set at p value.

Results and discussion

A total of 45 athletes from three different sports clubs interested in goalball in the XXX region were reached. Ten players were female, and fifteen players did not participate in the training regularly, so they were excluded from the study. One athlete was excluded due to an acute wrist injury. Two athletes did not come to the evaluation. In conclusion, seventeen male players (B1=6, B2=6, B3=5) participated in the study. Mean age of athletes is 20.82 (SD=3.94) years. The mean body mass index of the participants was 22.64 (SD=1.92) kg/m^2. It was noted that the players included in the study were interested in goalball for an average of 58.76±37.87

months. When the injury history of the players was taken, it was recorded that there was a total of 5 players with a history of mild orthopaedic injuries such as sprains and injuries. The demographic data of the players included in the study are presented in Table 1.

Table 1. Demographic data of athletes

		B1 (n=6)	B2 (n=6)	B3 (n=5)	Total (n=17)	p
**Age (year)**		22.66±4.67	19.60±2.30	20.0±4.14	20.82±3.94	0.329
**Cause of disability**	**Congenital**	6	6	5	17	0.378*
	**Postnatal**	0	0	0	0	
**Body mass index**		23.04±2.7	22.51±1.76	22.32±1.76	22.64±1.92	0.662
**Sports age (month)**		54.00±50.62	86.00±59.47	86.00±59.47	68.47±46.18	0.429
**Branch year (month)**		54.00±50.62	58.50±43.25	64.80±10.73	58.76±37.87	0.429
**Sports Injury History**		2	1	2	5	0.676*
**Throwing technique**	**Frontal**	4	3	4	11	0.580*
	**Spin**	2	3	1	6	

p=Kruskal Wallis test; p*=chi square test; n= number; B1. Athletes have limited perception of light and are not able to recognize any distance or any direction B2. Athletes can recognize shapes up to a certain visual field. B3. The visual field and visual acuity of the athletes are above a certain level.

Shoulder internal rotation joint position sense and the flamingo balance test were found to be different in the group with B1 visual acuity than in the group with B3 visual acuity (p=0.042* and 0.028 respectively) There was no difference between groups with B1-B2 visual acuity (p = 0.394 and p = 0.065) and between groups with B2-B3 visual acuity (p = 0.792 and p = 0.931), (Table 2) There was no difference in the area of the groups in terms of sensory tests (p > 0.05), (Table 3).

Table 2. Evaluation of sensory function according to visual acuity (reaction time and postural control)

		B1	B2	B3	p
**Reaction Time**		12.44±2.91	10.09±2.89	12.33±3.32	0.260
**Postural Control**	**Flamingo balance test**	4.91±1.74	2.62±1.74	2.21±0.29	0.028*
	**Functional reach test**	37.83±12.59	18.16±5.11	28.20±15.59	0.166

P=Kruskall-wallis test B1. Athletes have limited perception of light and are not able to recognize any distance or any direction B2. Athletes can recognize shapes up to a certain visual field. B3. The visual field and visual acuity of the athletes are above a certain level.

Table 3. Evaluation of senses (insert position sense, tender sense) according to visual acuity

			B1	B2	B3	p
**Shoulder Joint Position Sense**	**Internal Rotation**		2.0-3.94	4.5-5.61	8.40-3.84	0.042*
	**External Rotation**		2.83-3.81	4.5-5.61	4.60-6.98	0.263
**Sensory tests**	**Light Touch**	**1.Fingertip**	4.66-1.86	4.33-1.50	5.0-4.24	0.792
		**2. Fingertip**	4.83-1.60	4.16-1.32	5.0-4.24	0.662
		**3. Fingertip**	4.66-1.86	4.83-2.13	5.0-4.24	0.792
		**4. Fingertip**	4.66-1.86	4.66-1.36	5.0-4.24	0.792
		**5. Fingertip**	5.16-1.64	5.00-4.24	5.0-4.24	0.662
		**Palm**	6.66-1.96	6.50-3.61	6.52-3.65	0.662
	**Two-Point Discrimination**	**base of thumb**	2.83-0.34	2.97-0.56	2.83-0.56	0.804
		**ulnar side of hand**	2.89-0.38	2.68-0.22	2.83-0.56	0.257
		**Palm**	2.89-0.38	2.97-0.56	2.83-0.56	0.791

It was found that the players included in the study preferred the straight throw more than the rotary throw. The agility, shoulder internal and external joint position sense of the individuals who preferred the rotating shot were found to be better than the individuals who preferred the straight shot. It was found that sports age, branch year, and sense of reward did not affect

Throwing techniques (p>0.05), (Table 4). In addition, it was found that the level of visual acuity did not affect throwing techniques (p=0.691).

Table 4. Factors affecting throwing techniques.

			Throwing techniques (n=17)		
			**Frontal (n=11)**	**Spin (n=6)**	**p**
**Sports Age (month)**			57.81-36.62	88.00-58.62	0.202
**Branch Year**			57.81-36.62	60.50-4361	0.387
**Agility test**			11.85±1.90	11.08-3.83	0.034*
**Shoulder Internal Rotation Joint Position Sense**			7.50-6.89	1.81-1.77	<0.001**
**Shoulder External Rotation Joint Position Sense**			4.63-5.73	3.50-2.66	0.015*
**One Leg Standing Test**			3.23-1.87	3.44-1.95	0.971
**Functional Reach test**			27.09-15.21	29.83-11.94	0.608
**Sensory Tests**	**Light Touch**	**1. Fingertip**	4.45-2.73	5.00-2.38	0.642
		**2. Fingertip**	4.36-2.69	5.16-2.04	0.610
		**3. Fingertip**	4.45-2.73	5.50-2.58	0.880
		**4. Fingertip**	4.54-5.16	2.65-2.31	0.883
		**5. Fingertip**	5.33-2.06	4.63-2.69	0.757
		**Palm**	8.00-4.47	5.72-3.06	0.206
	**Two-point Discrimination**	**base of thumb**	2.99-0.27	2.87-074	0.072
		**ulnar side of hand**	2.92-0.29	2.59-0.47	0.184
		**Palm**	8.00-4.47	5.72-3.06	0.073

Mann Whitney u test, p*<0.04, p**<0.001

Goalball is a sport that requires agility, balance, and good use of body biomechanics [11]. Balance, joint position sense, and other sensory organs play an important role in the performance of sport-specific skills [16]. Visual acuity is the most important sensory function that provides data about the position of the body in order to provide agility and balance in sports skills [17]. When the reception of visual stimuli is limited, the data flow decreases. Visual acuity can affect goalball sports performance and ball shooting selection in general, or the lack of vision can be compensated by the athlete's improvement of other sensory functions [18]. Previous studies have shown that sport improves reflexes, senses, agility, balance, and

proprioceptive senses in general [19-21]. As in other paralympic sports, all athletes use eye patches in goalball. There is no obstacle classification in goalball [22]. However, visual impairment can develop adaptations in the somatosensory systems of visually impaired individuals to adapt to changing conditions. In the study of Kimyon and Ince, it was emphasized that the goal-shooting performances of the athletes with better visual acuity were higher [18]. Therefore, it can be said that visual acuity in goalball is important for athlete performance. An eye patch is used to provide equal opportunity to the athletes in the game of goalball. However, it is not clear how vision loss will create adaptations in other sensory functions related to performance. In sports with eyes closed, whether the group with low vision or the group with better visual acuity is more advantageous. Studies on this subject are also limited [18]. In a systematic review, the postural controls of visually impaired individuals were investigated. Although individuals with congenital blindness show structural changes in the cerebral cortex to compensate for the blindness, it has been reported that this does not lead to better postural control than individuals with typical development [23]. A few of the studies in the literature found that sports and exercise improved the postural control of blind individuals [15, 17, 19, 23].

In most studies in the literature, postural control assessments were performed with the eyes open. In our study, an eye patch was used for all evaluations. According to the postural control results of the study, it was seen that the shoulder joint position and static balance test results of the athlete with less visual acuity were better. This situation supports the judgment that the visual acuity of the athlete can affect her performance even if she uses an eye patch.

Goalball is a sport that requires quick manoeuvres from side to side and where agility and strength are important. Reaction time is a parameter that gives an idea about the functions of all the senses that directly affect the sport's skill. In the literature, the reaction time of visually impaired individuals is evaluated using the pro-agility test [24]. However, according to our

observations, there is a need to adapt this test to visually impaired athletes or to develop a new test specific to visually impaired athletes. According to a study, it is stated that visual acuity affects reaction time in sports because as visual acuity decreases, reaction time increases [25]. The important thing here is whether the evaluations are made using an eye patch. According to the evaluations made without using an eye patch, low vision constitutes a disadvantage. However, in our study, it was found that athletes with low visual acuity were more advantageous in terms of static balance, according to the evaluation results using an eye patch. There is no difference between the dynamic balance test results according to visual acuity. The shoulder internal rotation joint position was found to be more successful in the group with low visual acuity. It may have developed more in the group with less visual acuity since the internal rotation of the shoulder is used more during activities of daily living. The flow of information coming from the skin, muscles, joints, tendons, and tissues of the athlete and the athlete's movement patterns according to this information flow Therefore, the quality of the shoulder joint's proprioceptive sense will affect both shooting performance and the risk of injury. Joint position sense in athletes is a topic that has started to come to the fore in recent years, and there are limited studies in the literature [26]. Visual communication provides the most important data in the proprioceptive process. It is assumed that other perceptions develop more than normal due to visual impairment in visually impaired individuals; however, there are a limited number of studies on this information in the literature. In a thesis study, it was determined that the knee joint proprioception of visually impaired athletes was better than that of normal athletes. Similar to the results of our study, the sense of shoulder internal rotation joint position was found to be better in the group with lower visual acuity than in the other group. In another study conducted on visually impaired athletes in the literature, it has been reported that the knee joint position sense of visually impaired athletes is better than that of

sighted athletes, and it has been suggested that this deficiency can be treated by training the sighted athletes in the eyes closed position [10].

Although there are studies on balance and joint position in the literature, no study has been found about tactile sense. It is important to evaluate the sensitivity of mechanoreceptors in order to provide sufficient physiological evidence regarding the proprioceptive process. According to the results of our study, there was no difference between the presenting senses of the groups in terms of visual acuity. According to a study conducted on visually impaired children in the literature, it has been reported that visually impaired children use only tactile sense without the help of visual perception because they have lost their congenital vision; therefore, children with normal vision have better hand function test results [27]. In our study, however, no difference was found in terms of tactile, sensory, or visual acuity. Since the group evaluated in our study was made up of athletes, there may not have been a difference. Because it has been reported in previous studies that sports have positive effects on sensory function [24, 25].

Another variable examined in our study is throwing techniques. The main goal in goalball is to get the ball to the goal. It is possible for the athlete to deliver the ball to the goal with a technical throw. Rotary shooting and straight shooting are two different shooting techniques. Although there is not enough evidence in the literature, the rotational shot provides acceleration and makes the shot go harder [15]. Rotational shooting is a difficult technique with a high goal rate, requiring more biomechanics [15]. The results of our study also supported this information. According to our results, athletes who prefer rotational throwing have better shoulder internal rotation joint position sense and agility.

Limitation

It is a disadvantage of the study that not all athletes included in it are from the same club. There may be differences in training times and frequencies between clubs. The limited number of

visually impaired individuals who regularly practice goalball also prevented the study from being conducted with a large sample size.

Conclusion

The results of this study on scorers with different visual acuities are as follows: While athletes with low vision have better static balance and shoulder internal rotation joint position sense than athletes with better vision, there is no difference between dynamic balance results, agility, fixation sense, and shoulder external rotation joint position sense. Regarding the Throwing techniques s of the athletes, it was determined that the shoulder joint position sense and agility of the athletes who preferred the rotational shot were better. If the training of athletes with better visual acuity is supplemented with exercises with eyes closed, improvements in proprioceptive quality, postural control, and other sensory functions can be seen.

Acknowledgements

The authors acknowledge to athletes who contributed to this study.

Funding: This work was supported by The Scientific and Technological Research Council of XXX under Grant XXX. The funder did not assist in the preparation of the manuscript or exerted any influence on the manuscript's results.

Declarations

Conflict of Interest

The authors report there are no competing interests to declare.

Informed Consent: Informed consent was obtained from all individual participants included in the study.

References

1. de Haan EH, Dijkerman HC. Somatosensation in the brain: a theoretical re-evaluation and a new model. Trends Cogn Sci. 2020;24(7):529-41. doi: https://doi.org/10.1016/j.tics.2020.04.003.

2. Yamashiro K, Sato D, Onishi H, Yoshida T, Horiuchi Y, Nakazawa S, et al. Skill-specific changes in somatosensory-evoked potentials and reaction times in baseball players. Exp Brain Res. 2013;225:197-203. doi: https://doi.org/10.1007/s00221-012-3361-8.

3. Borghuis J, Hof AL, Lemmink KA. The importance of sensory-motor control in providing core stability: implications for measurement and training. Sports Med. 2008;38:893-916. doi: https://doi.org/10.2165/00007256-200838110-00002.

4. Adolphs R, Damasio H, Tranel D, Cooper G, Damasio AR. A Role for Somatosensory Cortices in the Visual Recognition of Emotion as Revealed by Three-Dimensional Lesion Mapping. J Neurosci. 2000;20(7):2683-90. doi: https://doi.org/10.1523/JNEUROSCI.20-07-02683.2000.

5. Gandevia S, McCloskey D, Burke D. Kinaesthetic signals and muscle contraction. Trends Neurosci. 1992;15(2):62-5. doi: https://doi.org/10.1016/0166-2236(92)90028-7.

6. Moody DB. Reaction Time as an Index of Sensory Function. In: Stebbins WC, editor. Animal Psychophysics: the design and conduct of sensory experiments. Boston, MA: Springer US; 1970. p. 277-302.

7. Riemann BL, Lephart SM. The sensorimotor system, part II: the role of proprioception in motor control and functional joint stability. J Athl Train. 2002;37(1):80-4.
8. Walicka-Cupryś K, Rachwał M, Guzik A, Piwoński P. Body Balance of Children and Youths with Visual Impairment (Pilot Study). Int J Environ Res Public Health. 2022;19(17):11095. doi: https://doi.org/10.3390/ijerph191711095. PubMed PMID: doi:10.3390/ijerph191711095.
9. Voss P, Collignon O, Lassonde M, Lepore F. Adaptation to sensory loss. WIREs Cogn Sci. 2011;2(2):238. doi: https://doi.org/10.1002/wcs.139.
10. Şilil MK, Sargın S, Atik A, Meriç G, Özer M, Akseki D. Comparison of Knee Proprioception Between Blind and Healthy Sportsmen. Orthop J Sports Med. 2014;2(11_suppl3). doi: https://doi.org/10.1177/2325967114S00177.
11. Bednarczuk G, Molik B, Morgulec-Adamowicz N, Kosmol A, Wiszomirska I, Rutkowska I, et al. Static balance of visually impaired paralympic goalball players. Int J Sports Sci Coach. 2017;12(5):611-7. doi: https://doi.org/10.1177/1747954117727791.
12. Dover GC, Kaminski TW, Meister K, Powers ME, Horodyski M. Assessment of Shoulder Proprioception in the Female Softball Athlete. The American Journal of Sports Medicine. 2003;31(3):431-7. doi: https://www.doi.org/10.1177/03635465030310031801. PubMed PMID: 12750139.
13. Zimney K, Dendinger G, Engel M, Mitzel J. Comparison of reliability and efficiency of two modified two-point discrimination tests and two-point estimation tactile acuity test. Physiotherapy Theory and Practice. 2022;38(1):235-44. doi: https://doi.org/10.1080/09593985.2020.1719563.
14. Tsigilis N, Douda H, Tokmakidis SP. Test-Retest Reliability of the Eurofit Test Battery Administered to University Students. Perceptual and Motor Skills. 2002;95(3_suppl):1295-300. doi: https://doi.org/10.2466/pms.2002.95.3f.1295. PubMed PMID: 12578274.

15. Makaracı Y, Nas K, Pamuk Ö, Aydemir M. Relationships among postural stability, physical fitness, and shooting accuracy in Olympic female goalball players. J Exerc Rehabil. 2022;18(5):308-17. doi: https://www.doi.org/10.12965/jer.2244376.188.

16. Molik B, Morgulec-Adamowicz N, Kosmol A, Perkowski K, Bednarczuk G, Skowroński W, et al. Game performance evaluation in male goalball players. J Hum Kinet. 2015;48:43-51. doi: https://www.doi.org/10.1515/hukin-2015-0090.

17. Santos CdN, Carvalho TLd, Felício LR, Mainenti MRM, Vigário PdS. Postural control in athletes with different degrees of visual impairment. J Phys Educ. 2018;29. doi: https://doi.org/10.4025/jphyseduc.v29i1.2936.

18. Kimyon B, Ince G. The Comparison of Physical Fitness, Anthropometric Characteristics, and Visual Acuity of Goalball Players with Their Right Shot Performance. J Vis Impair Blind. 2020;114(6):516-30. doi: https://doi.org/10.1177/0145482X20972501.

19. Taskın C, Biçer Y. The effect of an eight-week proprioception training program on agility, quickness and acceleration. Turk J Sport Exer. 2015;17(2):26-30. doi: https://doi.org/10.15314/tjse.81867.

20. Ashton-Miller JA, Wojtys EM, Huston LJ, Fry-Welch D. Can proprioception really be improved by exercises? Knee Surg Sports Traumatol Arthrosc. 2001;9(3):128-36. Epub 2001/06/26. doi: https://doi.org/10.1007/s001670100208. PubMed PMID: 11420785.

21. Ma X, Lu L, Zhou Z, Sun W, Chen Y, Dai G, et al. Correlations of strength, proprioception, and tactile sensation to return-to-sports readiness among patients with anterior cruciate ligament reconstruction. Front Physiol. 2022;13. doi: https://www.doi.org/10.3389/fphys.2022.1046141.

22. Floyd B, Mowling C. Goalball: Strategies for Teaching One Paralympic Sport in Inclusive Physical Education. ASAHPERD Journal. 2019;39(2):22-7.

23. Parreira RB, Grecco LAC, Oliveira CS. Postural control in blind individuals: A systematic review. Gait Posture. 2017;57:161-7. doi: https://doi.org/10.1016/j.gaitpost.2017.06.008.

24. Bıyıklı T, Güler U, Kuruoğlu YA, Akgül E, Çetinkaya S, Boyaci A. The evaluation of physical fitness improvement after 8 weeks individual strength and conditioning program on b1 turkish national football team. European Journal of Physical Education and Sport Science. 2018;4(5):114-26. doi: https://doi.org/10.5281/zenodo.1246030.

25. Stavropoulou G, Stavropoulos N. Association between team sports and anxiety with reaction time of individuals with visual impairment versus individuals with normal vision. Trends in Sport Sciences. 2021;28(2):117-25. doi: https://www.doi.org/10.23829/TSS.2021.28.2-5.

26. Şilil MK. Görme engelli sporcularda diz eklemi propriyosepsiyon ölçümlerinin gören sporcularla karşılaştırılması [Master Thesis]. Balıkesir: Balikesir University; 2014.

27. Atasavun Uysal S, Akı E. Konjenital görme engelli çocukların el fonksiyonlarının gören yaşıtları ile karşılaştırılması. Physiotherapy Rehabilitation. 2009;20(2):76-82.

Supporting information

Figure 1. Sensory test (two-point discrimination)

Figure 2. Sensory function test (reaction time)

Table 1. Demographic data of athletes.

Table 2. Evaluation of sensory function according to visual acuity (reaction time and postural control)

Table 3. Evaluation of senses (insert position sense, tender sense) according to visual acuity.

Table 4. Factors affecting throwing techniques.

Figure1

Click here to access/download;Figure;Figure1.jpg

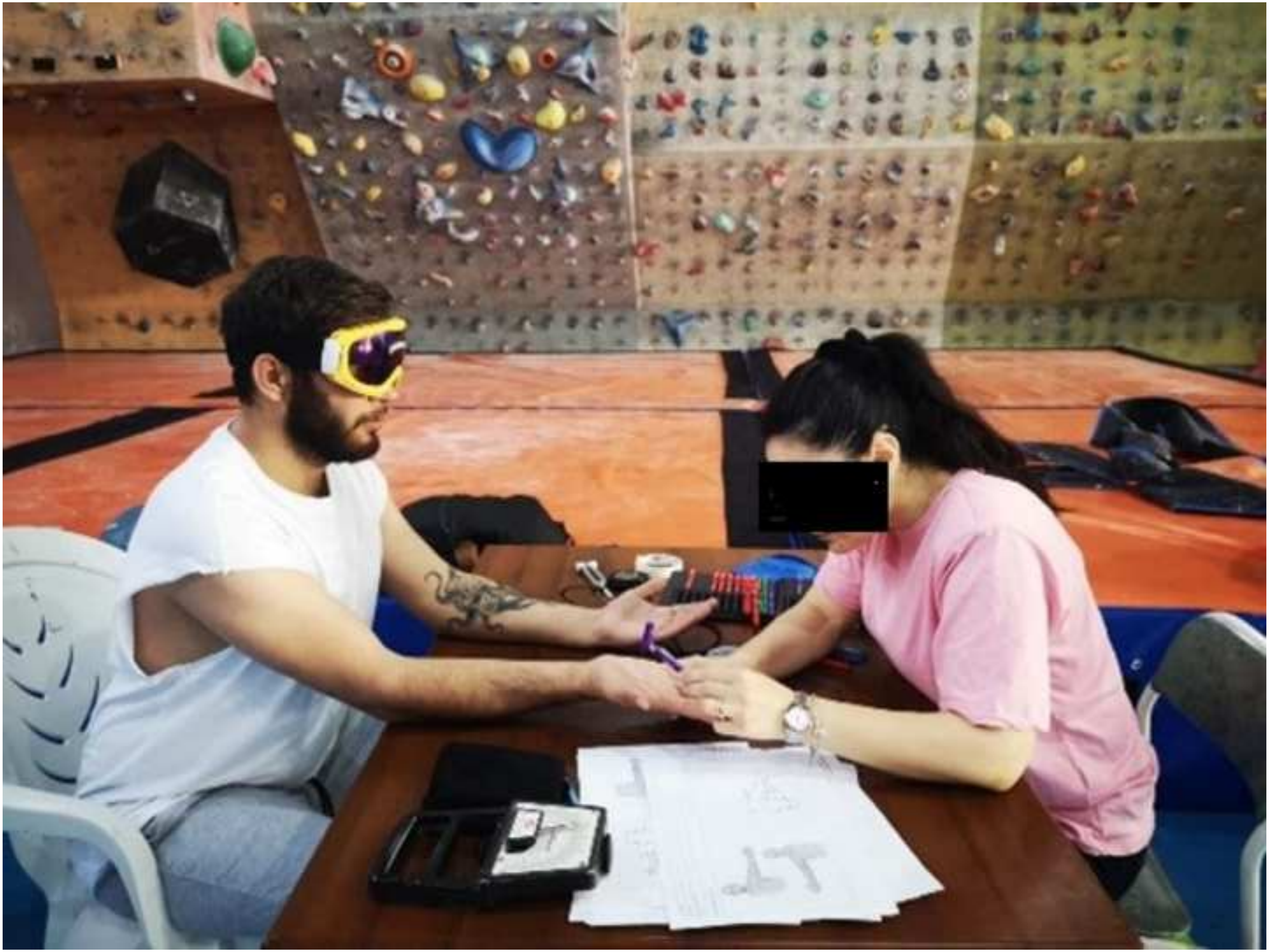

Figure2

Click here to access/download;Figure;Figure2.jpg

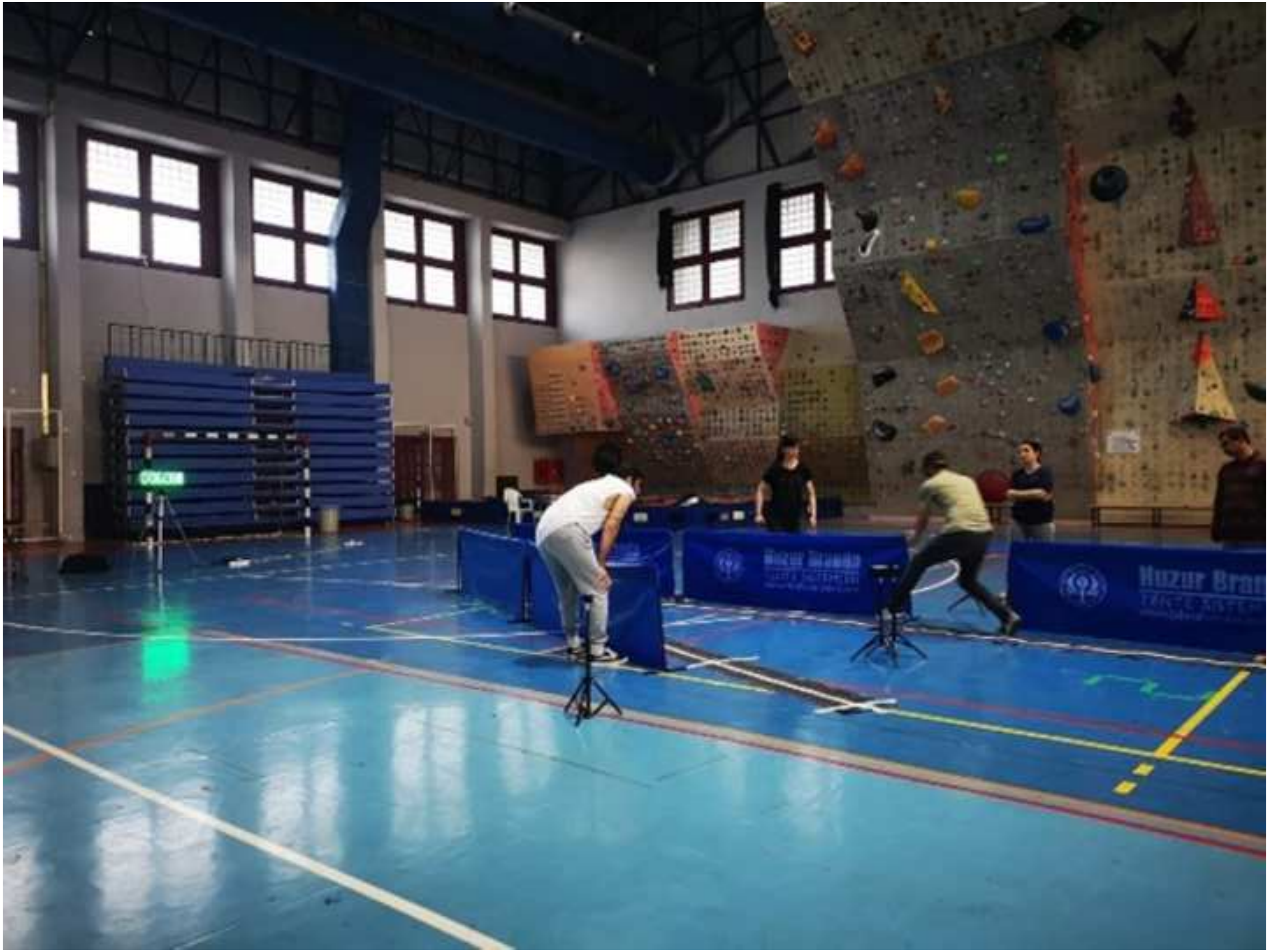

Click here to access/download
Supporting Information
Supporting information.docx

Ethics approval english translation

Click here to access/download
Supporting Information
etik onay belgesi.tr.en.docx
